# Automated detection of enlarged extraocular muscle in Graves’ ophthalmopathy with computed tomography and deep neural network

**DOI:** 10.1038/s41598-022-20279-4

**Published:** 2022-09-26

**Authors:** Kaori Hanai, Hitoshi Tabuchi, Daisuke Nagasato, Mao Tanabe, Hiroki Masumoto, Sakurako Miya, Natsuno Nishio, Hirohiko Nakamura, Masato Hashimoto

**Affiliations:** 1 Department of Ophthalmology, Nakamura Memorial Hospital, Sapporo, Japan; 2 Department of Ophthalmology, Tsukazaki Hospital, 68-1 Aboshi-Waku, Himeji City, Hyogo Prefecture 671-1227 Japan; 3 Department of Technology and Design Thinking for Medicine, Hiroshima University Graduate School, Hiroshima, Japan; 4 Department of Ophthalmology, Institute of Biomedical Sciences, Tokushima University Graduate School, Tokushima, Japan; 5 Department of Neurosurgery, Nakamura Memorial Hospital, Sapporo, Japan

**Keywords:** Diseases, Medical research

## Abstract

This study aimed to develop a diagnostic software system to evaluate the enlarged extraocular muscles (EEM) in patients with Graves’ ophthalmopathy (GO) by a deep neural network.This prospective observational study involved 371 participants (199 EEM patients with GO and 172 controls with normal extraocular muscles) whose extraocular muscles were examined with orbital coronal computed tomography. When at least one rectus muscle (right or left superior, inferior, medial, or lateral) in the patients was 4.0 mm or larger, it was classified as an EEM patient with GO. We used 222 images of the data from patients as the training data, 74 images as the validation test data, and 75 images as the test data to “train” the deep neural network to judge the thickness of the extraocular muscles on computed tomography. We then validated the performance of the network. In the test data, the area under the curve was 0.946 (95% confidence interval (CI) 0.894–0.998), and receiver operating characteristic analysis demonstrated 92.5% (95% CI 0.796–0.984) sensitivity and 88.6% (95% CI 0.733–0.968) specificity. The results suggest that the deep learning system with the deep neural network can detect EEM in patients with GO.

## Introduction

Graves’ ophthalmopathy (GO) is a chronic autoimmune disorder that affects the retrobulbar tissues and extraocular muscles with strong etiological links to autoimmune thyroid disease. Extraocular muscle dysfunction reportedly occurs in approximately 40%–60% of patients with GO in actual clinical practice^1,2^ and has significant negative effects on the quality of life^3^. Early detection of extraocular muscle abnormalities on orbital imaging might thus be necessary for managing thyroid myopathy successfully. In actual clinical practice, orbital imaging is not likely to be performed unless the patient complains of double vision. Additionally, radiologists may not always be available to interpret the findings, especially in regions with a shortage of doctors^4,5^. In some regions of developing countries, facilities for adequate imaging might be scarcer than radiologists.

Supervised machine learning systems, known as neural networks, have been applied to medical research^6^. Many studies on the diagnostic and classification performance of deep learning (DL) systems with CT images have been conducted^7–13^. However, to the best of our knowledge, there has not been a report in which DL systems have classified enlarged extraocular muscle (EEM) images in patients with GO and normal extraocular muscle (NEM) images in normal subjects using CT images.

This research aimed to develop a diagnostic software system in which a DL system could evaluate the EEM in patients with GO with orbital CT images.

## Results

We used EEM images from 199 patients (56 men and 143 women) with GO (mean age, 55.9 ± 13.7 years) and NEM images from 172 controls (40 men and 132 women; mean age, 52.6 ± 18.4 years) in this analysis. We found no significant differences in age (p = 0.21) or gender (p = 0.85) between the two groups (Table 1).

**Table 1:** Participant characteristics.

Characteristics	EEM	NEM	p-value
Number of participants	199	172	
Age (years)	55.9 ± 13.7	52.6 ± 18.4	0.21 (unpaired *t*-test)
Gender (male/female)	56/143	40/132	0.85 (Fisher’s exact test)

Unless otherwise indicated, these data are expressed as means ± standard deviations.

*EEM* enlarged extraocular muscle, *NEM* normal extraocular muscle.

Table 2 shows the right and left superior, inferior, medial, and lateral rectus muscles in the two groups. All right or left rectus muscle thicknesses differed significantly between the two groups (each p < 0.001).

**Table 2:** The difference in the maximum diameter between enlarged extraocular muscle (EEM) and normal extraocular muscle (NEM).

Eye	EEM	NEM	p-value
**Right**			
SRM	4.33 ± 1.47	3.06 ± 0.57	< 0.001
IRM	4.62 ± 1.44	3.19 ± 0.51	< 0.001
MRM	4.16 ± 1.22	3.24 ± 0.49	< 0.001
LRM	3.20 ± 1.21	2.76 ± 0.52	< 0.001
**Left**			
SRM	4.17 ± 1.36	2.87 ± 0.56	< 0.001
IRM	4.69 ± 1.37	3.19 ± 0.50	< 0.001
MRM	4.09 ± 1.05	3.27 ± 0.46	< 0.001
LRM	3.09 ± 0.97	2.60 ± 0.48	< 0.001

Unless otherwise indicated, the EEM and NEM data are expressed as means ± standard deviations.

*EEM* enlarged extraocular muscle, *IRM* inferior rectus muscle, *LRM* lateral rectus muscle, *MRM* medial rectus muscle, *NEM* normal extraocular muscle, *SRM* superior rectus muscle.

**Figure 1:**
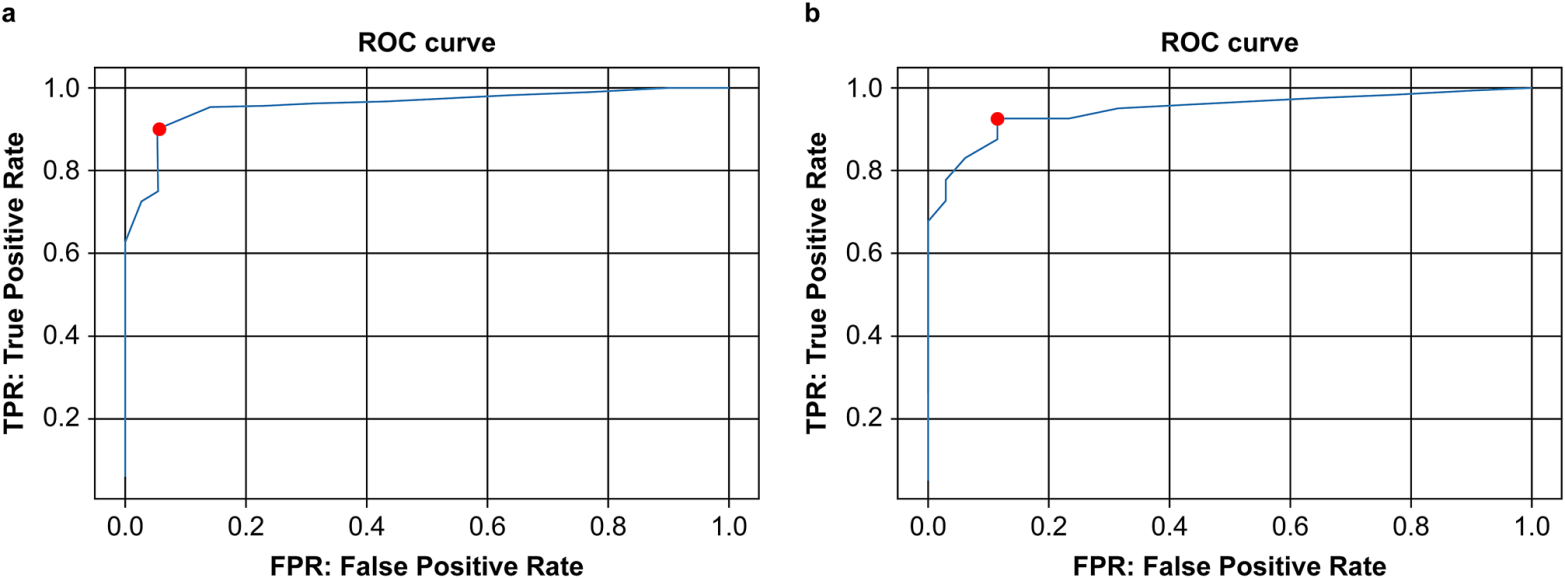
(**a**) Receiver operating characteristic (ROC) curve for the validation data. The area under the curve (AUC) for diagnosis by the neural network was 0.953, and ROC analysis revealed 89.7% sensitivity and 94.3% specificity. (**b**) ROC curve for the test data. The AUC for diagnosis by the neural network was 0.946, and ROC analysis revealed 92.5% sensitivity and 88.6% specificity.

In the test data, the area under the curve (AUC) diagnosis by the neural network was 0.946 (95% confidence interval [CI] 0.894–0.998), and receiver operating characteristic (ROC) analysis demonstrated 92.5% (95% CI 0.796–0.984) sensitivity and 88.6% (95% CI 0.733–0.968) specificity (Fig. 1). For the test data, 276.2 s was needed to analyze the CT scans of 75 patients (3.6 s/patient).

**Figure 2:**
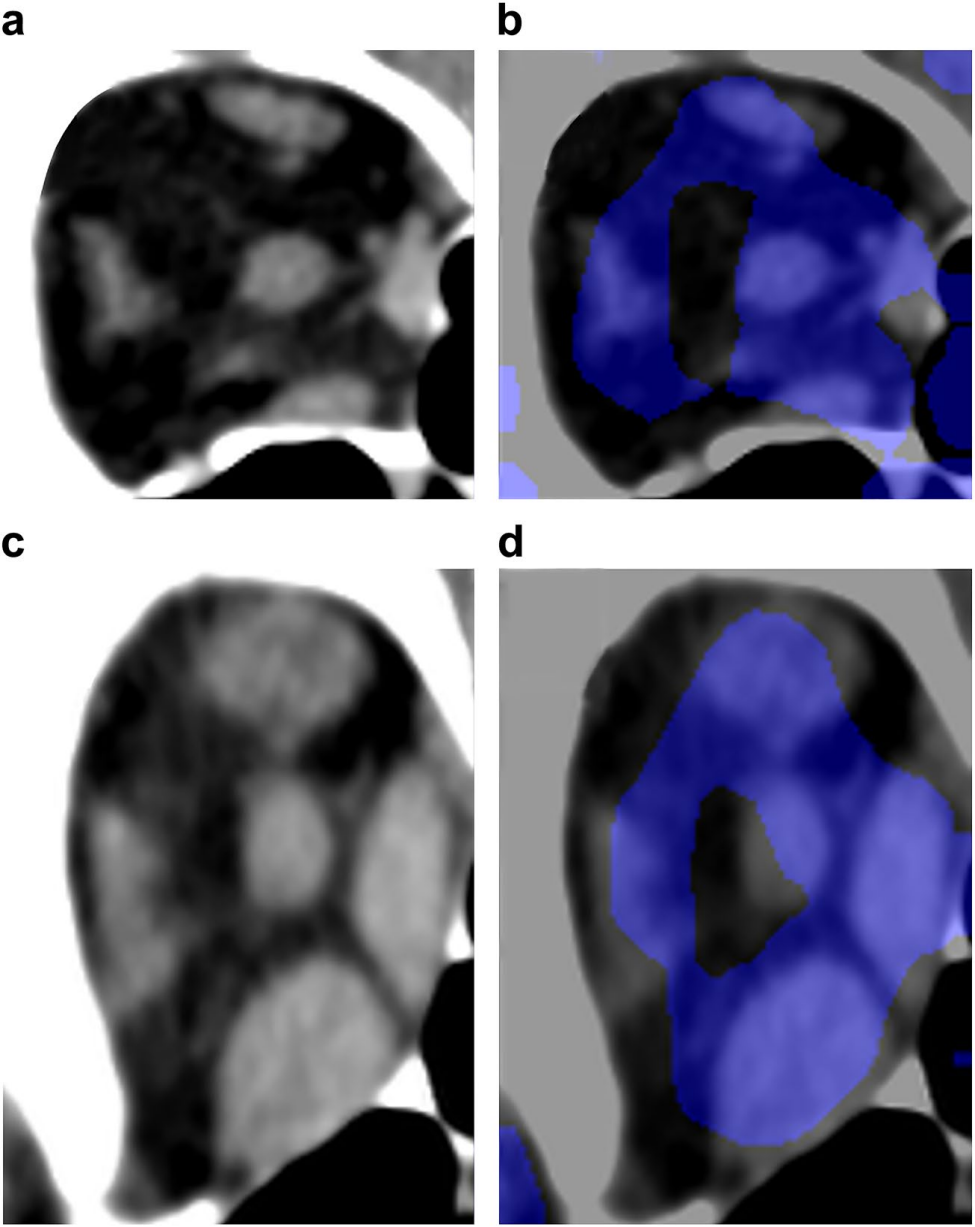
The computed tomographic (CT) slice image (**a**) and the heat map (**b**) for a healthy participant. The CT slice image (**c**) and the heat map (**d**) for a patient with Graves’ ophthalmopathy. Blue coloration indicates the strength of deep neural network attention. The color intensity is high at the area of the rectus muscles on the orbital coronal CT image. The deep neural network classifies the extraocular muscles as enlarged in the patient with Graves’ ophthalmopathy and as normal in the controls, focusing on the rectus muscles.

Figure 2 shows composite images where the representative orbital CT images of patients with GO and healthy participants were layered with the heat maps. The right and left rectus muscles in the orbital CT images are displayed in blue, indicating the parts of the image where the DL model focuses on distinguishing between EEM and NEM.

## Discussion

We investigated whether a DL system could evaluate EEM in patients with GO. This system was able to classify both EEM and NEM with high AUCs, sensitivity, and specificity, indicating that the system distinguished images as belonging to participants with EEM or those with NEM on orbital CT images with nearly the same level of accuracy as that of doctors.

Our study defined the 4-mm thickness of the extraocular muscle diameter as abnormal. This cutoff value was determined based on previous reports of Dutton showing NEM thickness. However, Ozgen et al.^14^ reported that mean maximum diameters of the extraocular muscles measured using conventional CT were MR 4.2 (range 3.3–5.0) mm, LR 3.3 (1.7–4.8) mm, SR 4.6 (range 3.2–6.1) mm, and IR 4.8 (range 3.2–6.5) mm. In their study, they used conventional CT. In this CT, individual variations in the chin-up posture of participants during coronal section imaging were observed, which may enhance the variability of extraocular muscle thickness. Conversely, spiral CT is used in our study. Spiral CT is created by reconstructing horizontal cross-sectional images, which are captured at the same angle due to participants’ constant posture during imaging. Therefore, our results showed less variation in extraocular muscle thickness in the control group compared to the findings of Ozgen et al. Therefore, we assumed that our extraocular muscle thickness results were consistent with Dutton’s, with an average thickness of less than 4 mm for each extraocular muscle.

A nationwide survey of patients with GO in the United Kingdom revealed delays in diagnosis, wide variability of access to specialist centers, appropriate treatment, and overall low patient satisfaction with treatment^15^. The same study revealed that only 25% of patients had referrals to a specialist GO clinic and that referrals were typically late. In several studies on general health-related questionnaires about quality of life among patients with GO, the scores of these patients were lower than those of the healthy reference population^16,17^. Gerding et al. reported that quality-of-life scores among patients with GO were worse than those in patients with diabetes, emphysema, or heart failure^16^. In approximately 70% of adults with Graves’ hyperthyroidism, magnetic resonance imaging or CT scanning reveals EEM^18^. Physicians thus need to monitor patients for ocular signs, including lid edema, lid retraction, and proptosis on visual inspection, and EEM, as demonstrated on orbital imaging, in patients with Graves’ hyperthyroidism. We consider that early detection and treatment of thyroid myopathy may become possible if the DL software system evaluating EEM in GO plays a supporting role in the actual clinical practice.

The modified clinical activity score (CAS) is currently the most widely used index to determine the active phase of inflammation in GO^19^. However, a recent study of GO indicated that the CAS may not reflect the inflammatory activity of myopathy, especially in mild to moderate GO with low NOSPECS scores (no sign of thyroid disease, only eyelid signs, soft tissue involvement, proptosis, extraocular motility restriction, corneal involvement, and sight loss). This system classifies the clinical severity of GO with low exophthalmos values^20,21^. Nagy et al. reported that EEM does not imply the presence of edematous swelling, and the severity of diplopia is unrelated to the degree of ocular congestion and edema^20^. Kim et al. reported that 44.4% of patients with GO and progressive diplopia had low CASs and no typical symptoms of inflammation^21^. These findings may have arisen because the CAS reflects primarily ocular muscle involvement and acute orbital congestion, which represents inflammatory changes within orbital connective and adipose tissues. Ophthalmologists thus must detect EEM early in the course of GO.

In our heat maps showing the focus of DL, color intensity surrounding the rectus muscles on the orbital CT images increased. The areas in the orbital CT images that the DL system focused on were consistent with those that ophthalmologists focus on when using CT images, they confirm EEM. In other words, the generated heat maps suggest that DL systems can accurately detect EEM associated with GO on the orbital CT images. Our DL software system may be helpful in the ophthalmological assessment of patients with GO.

Our system had several limitations. First, our study was conducted within a single facility, and the model’s robustness must be evaluated prospectively with data from multiple facilities. Second, from the perspective of radiation exposure to the participants, images with a slice thickness of 2 mm were used during CT imaging in this study. Using images with finer slice thickness may improve accuracy. Third, the judgment of EEM was based on measurements of the thickness of the muscles on two-dimensional CT images. The muscles’ volumetric measurement must be evaluated on three-dimensional CT or magnetic resonance images. Finally, DL’s performance and versatility should be evaluated extensively with larger samples and more images.

In conclusion, our results indicate that our DL system and orbital coronal CT had high accuracy for detecting EEM in GO. DL systems to screen orbital coronal CT images may yield useful information about early treatment for EEM patients with GO.

## Methods

### Patients

This prospective observational study complied with the Declaration of Helsinki. The study protocol followed the ethics committees of Nakamura Memorial and Tsukazaki Hospital. The patients provided written informed consent for the publication of this study and accompanying images. All experimental protocols were approved by the licensing committees of these hospitals.

In this study, we examined data from patients with GO and healthy normal subjects who had orbital CT scans at Nakamura Memorial Hospital between February 2017 and November 2019. An experienced neuro-ophthalmologist diagnosed GO using Bartley and Gorman's criteria^22^. Patients with orbital tumors, blowout fractures, immunoglobulin G4-associated ophthalmopathy, or idiopathic orbital inflammation were excluded from this study.

**Figure 3:**
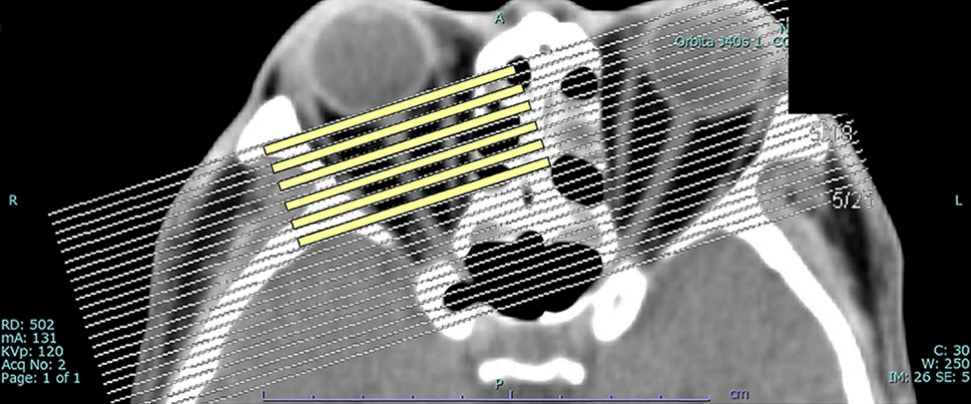
Coronal scans in a paraxial plane 90° to the orbital axis were reconstructed from the axial scans (**a**). Sequential six slices (2-mm thickness) from the posterior margin of the globe toward the orbital apex on the coronal scans were used (**b**).

Extraocular muscles were analyzed with orbital images obtained using a whole-body CT system (SOMATOM Definition AS+; Siemens, Erlangen, Germany) without contrast. Axial scans were obtained at an angle of − 10° to − 15° to the orbitomeatal line, and coronal scans in a paraxial plane 90° to the orbital axis were reconstructed from the axial scans (slice thickness, 2 mm). We measured the diameter of all rectus muscles shown on six slices from the globe’s posterior margin to the orbital apex (Fig. 3). The maximum diameter was defined as the thickest diameter of each muscle on the six slices. The spindle-like spreading of the rectus muscles without tendon involvement was identified morphologically as EEM^23^. Diameters of the superior, inferior, medial, and lateral rectus muscles were measured on coronal scans. The inferior and superior oblique muscles were excluded because their course is oblique to the coronal plane.

Anatomically, the rectus muscles are typically 2.5–4.0 mm thick at the midpoint^24^. Therefore, we classified rectus muscles > 4.0 mm thick as enlarged. On this basis, this study involved 371 participants (199 patients with EEM and 172 controls with NEM). All 199 EEM patients were diagnosed with GO.

### The DL model and its training

**Figure 4:**
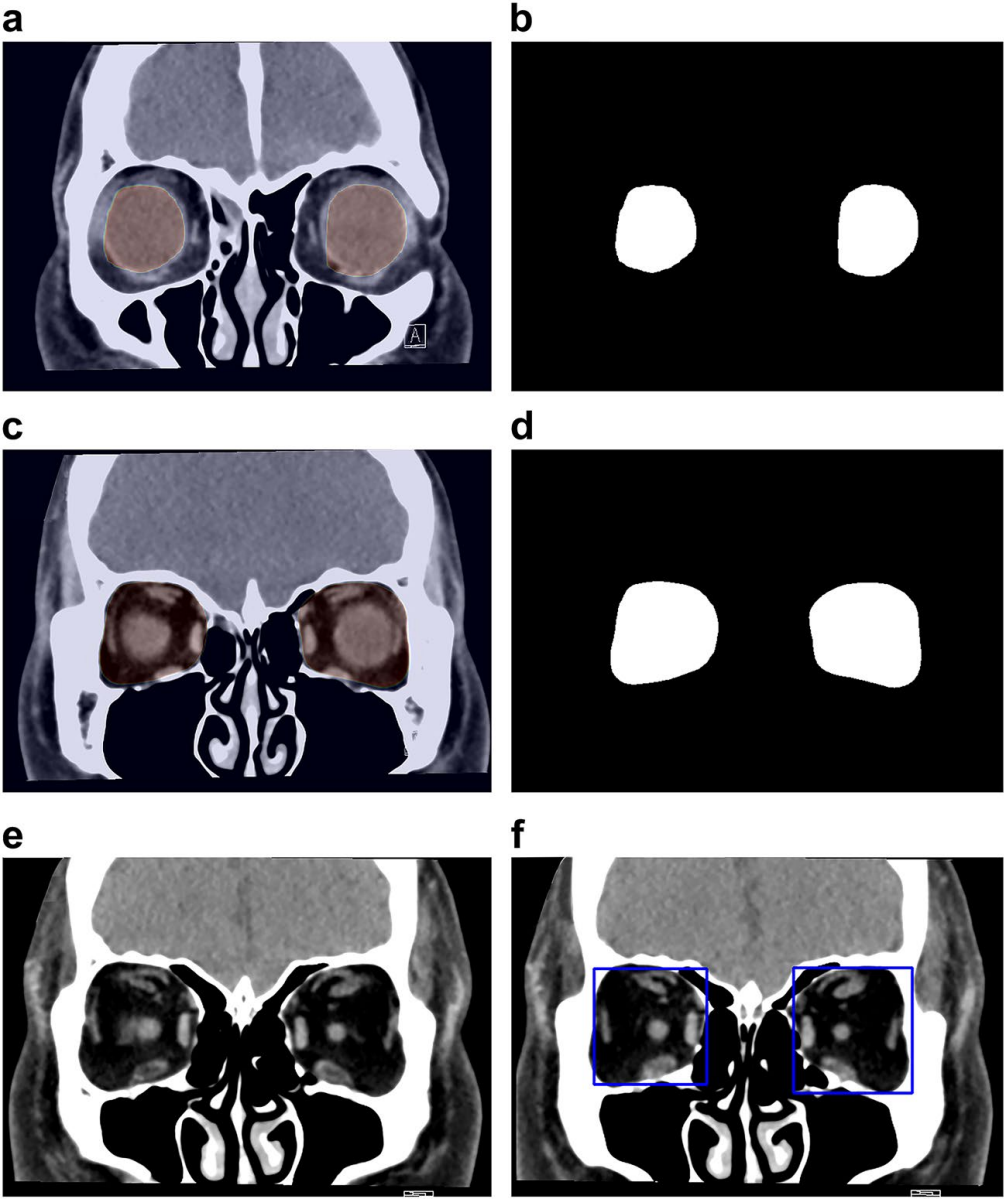
The coronal slice (**a**) and the result (**b**) used for the segmentation of the eyeball. The coronal slice (**c**) and the result (**d**) were used for the orbit segmentation. The coronal slice (**e**) and region of interest (the area inside the blue squares) (**f**) used when Residual Network-50 recognized the retrobulbar region from (**b**) and (**d**).

The DL algorithm consists of four main processes: (1) extraction of the retrobulbar region from the CT image; (2) trimming of the orbital area on the CT image; (3) classification of the presence or absence of hypertrophied extraocular muscle; and (4) evaluation of extraocular muscle abnormality in GO. For down-sampling and up-sampling, the neural network architecture for segmentation was obtained through Residual Network-50^25^ (Supplementary Fig. S1). First, the globe was segmented on coronal CT slices, and the orbital region posterior to the segmented globe was segmented and trimmed using Residual Network-50 (Fig. 4). The code is provided in the supplemental data. Next, all rectus muscles judged by the neuro-ophthalmologist to be abnormal on coronal CT were tagged. For classification, we used the Visual Geometry Group-16^26^ as the neural network and trained the DL system using the tag (Supplementary Fig. S2). The neural network generates the probability for each slice’s category (e.g., 0.1 for normal and 0.9 for abnormal). If the probability of “abnormal” exceeds a certain threshold, the slice is considered abnormal. We calculated this threshold from the validation data. Additionally, we calculated the proportion of slices considered abnormal by the neural network. If the proportion exceeded a certain threshold, the CT data as a whole was judged to reveal extraocular muscle abnormalities.

For all model training, the loss function was the sum of binary cross-entropy and dice loss, batch size was 16, and epochs was 100. These details are included in the supplemental codes.

For training data, we used coronal scans from 120 patients with EEM and 102 controls with NEM; for validation data, we used scans from 39 patients with EEM and 35 controls with NEM; and for test data, we used scans from 40 patients with EEM and 35 controls with NEM.

### Statistical analysis

We used Fisher’s exact test and the unpaired *t*-test to compare differences between EEM and NEM. We constructed ROC curves and the proportion of CT slices judged as abnormal by the neural network based on the diagnostic imaging data. Then, we calculated the AUC of the ROC curve, the point at which the ROC curve was closest to the upper left (100% sensitivity, 100% specificity), and the sensitivity and specificity. The 95% CI of the AUC was calculated assuming a normal distribution^27^; the Clopper–Pearson method was used to calculate the 95% CIs for sensitivity and specificity^28^.

All statistical analyses were performed using the Python library SciPy (https://www.scipy.org/). Significance was expressed by p < 0.05.

### Heat map

The two main types of explainability in machine learning technology are intrinsic explainability and post hoc explainability^29^. In this study, we used Score-CAM (score-weighted class activation mapping), a type of post hoc visual explanation method^30^, to construct heat maps for indicating the areas where images in the convolutional neural network were focused. The target layer was the block5_conv2 layer of Visual Geometry Group-16. The heat maps revealed that the model focused more on the blue parts of the image.

## Supplementary Information

Supplementary Legends.

Supplementary Figure S1.

Supplementary Figure S2.

## Data Availability

The CT images and the image data sets used in this study are available upon reasonable request from the corresponding authors.
