# Supplementary material for: Automated detection of enlarged extraocular muscle in Graves’ ophthalmopathy with computed tomography and deep neural network
Source: Sci Rep. 2022 Sep 26;12:16036. doi: 10.1038/s41598-022-20279-4 (PMC9512911; doi:10.1038/s41598-022-20279-4)
Supplement: Supplementary file 1 — Supplementary Legends. [file 41598_2022_20279_MOESM1_ESM.docx]

# **Supplementary Data**

**Supplementary Figure S1. The overall architecture of the Residual Network-50 (ResNet-50) model.** The ResNet-50 architecture consists of multiple convolution and identity blocks. To bypass multiple convolution layers, “Skip connections” were included in the blocks. Another feature of ResNet-50 is the absence of max-pooling, fully connected, and dropout layers included in Visual Geometry Group-16.

**Supplementary Figure S2.** The overall architecture of the Visual Geometry Group-16 model. Visual Geometry Group-16 consists of five blocks and three fully connected layers. Each block consists of several convolution layers followed by a max-pooling layer. The flattened layer follows block 5 with two fully connected layers, and a binary classification is performed. The deep neural network used the ImageNet parameter as the default weight for blocks 1–4.
