# Supplementary figures and images for: Automated detection of enlarged extraocular muscle in Graves’ ophthalmopathy with computed tomography and deep neural network
Source: Sci Rep. 2022 Sep 26;12:16036. doi: 10.1038/s41598-022-20279-4 (PMC9512911; doi:10.1038/s41598-022-20279-4)

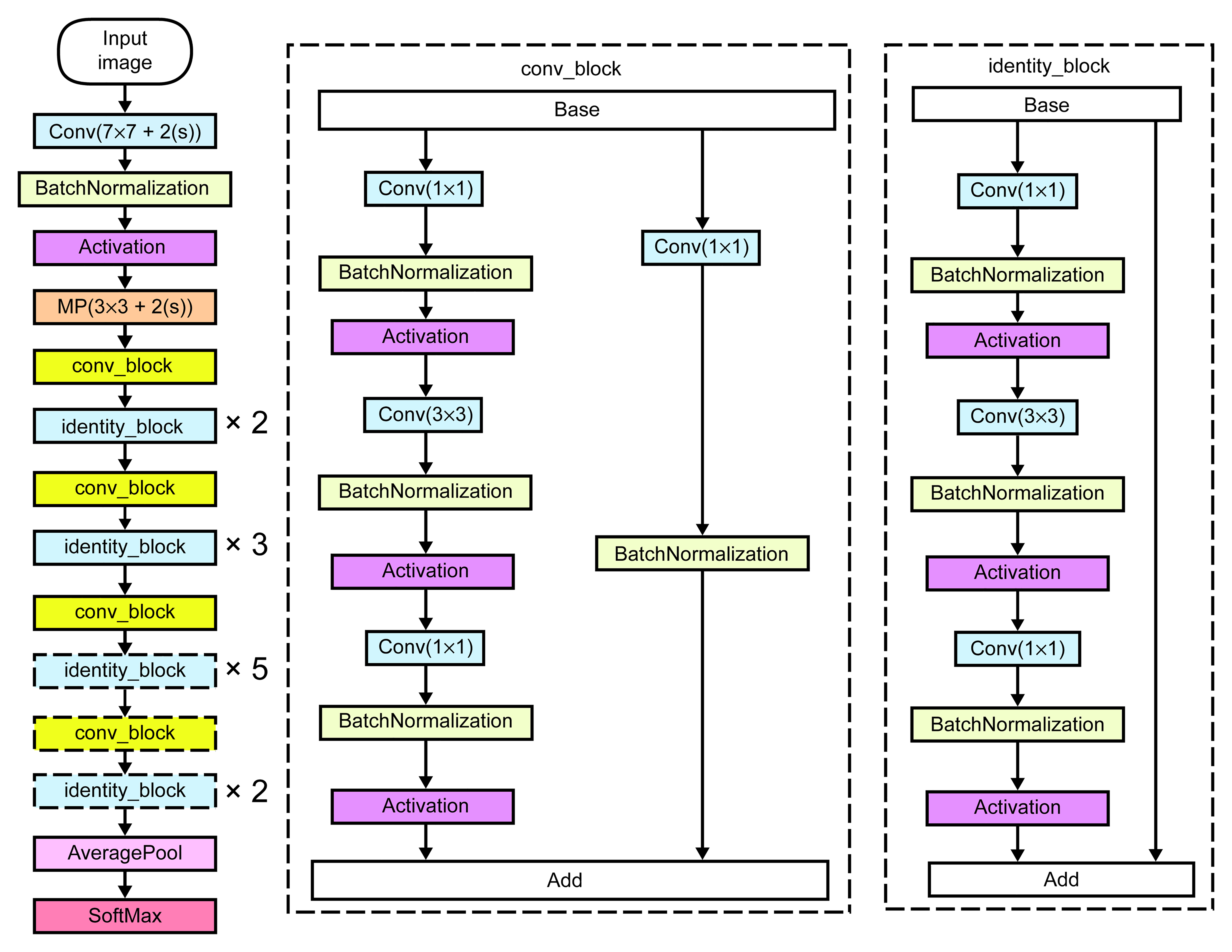

Supplement: Supplementary file 2 — Supplementary Figure S1. [file 41598_2022_20279_MOESM2_ESM.tif]

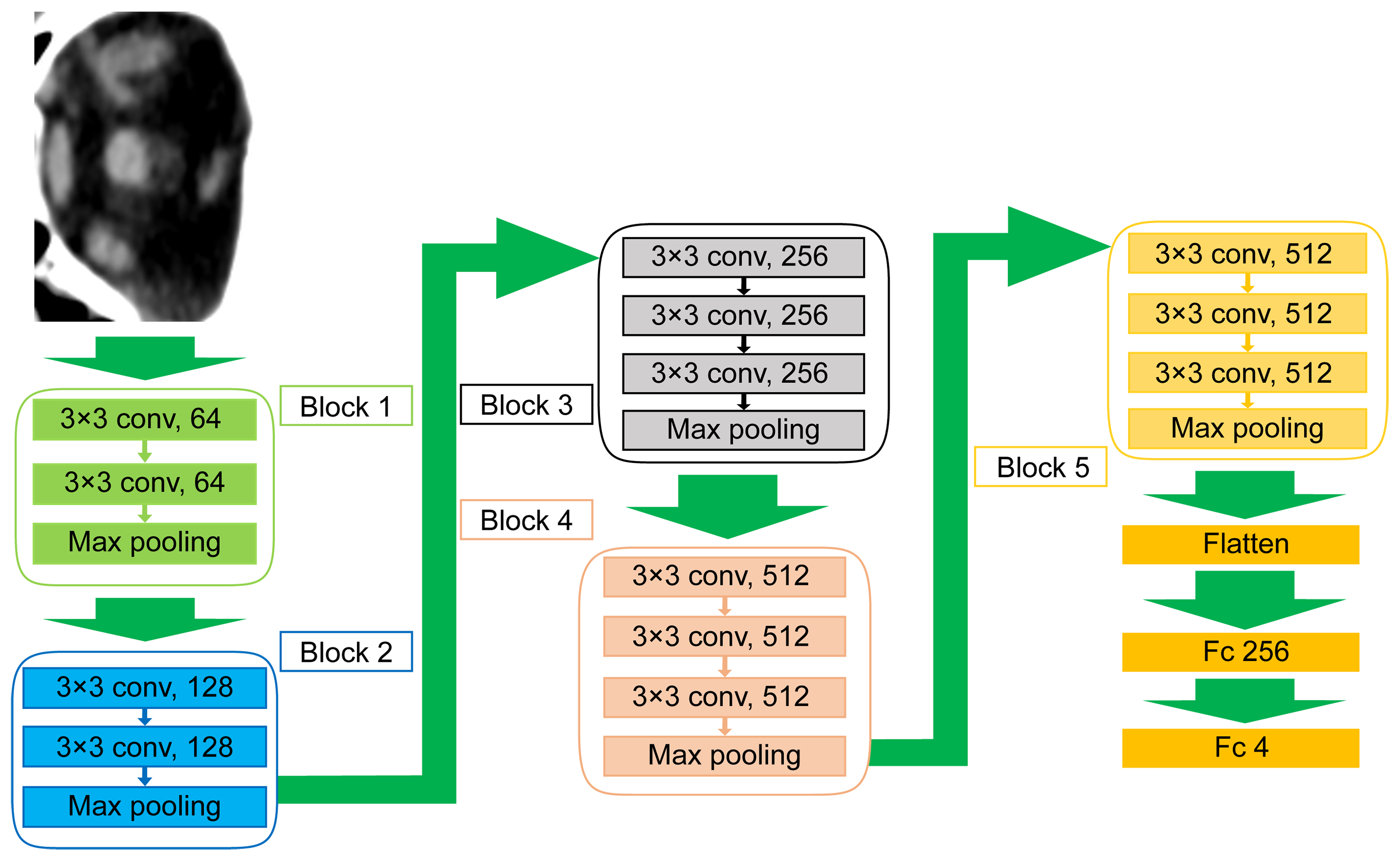

Supplement: Supplementary file 3 — Supplementary Figure S2. [file 41598_2022_20279_MOESM3_ESM.tif]
